# How the Avengers assemble: Ecological modelling of effective cast sizes for movies

**DOI:** 10.1371/journal.pone.0223833

**Published:** 2020-02-26

**Authors:** Matthew Roughan, Lewis Mitchell, Tobin South

**Affiliations:** ARC Centre of Excellence for Mathematical & Statistical Frontiers (ACEMS), School of Mathematical Sciences, University of Adelaide, Australia; Indiana University, UNITED STATES

## Abstract

The number of characters in a movie is an important feature. However, it is non-trivial to measure directly, for example naive metrics such as the number of credited characters vary wildly. Here, we show that a metric based on the notion of ecological diversity as expressed through a Shannon-entropy based metric can characterise the number of characters in a movie, and is useful in taxonomic classification. We also show how the metric can be generalised using Jensen-Shannon divergence to provide a measure of the similarity of characters appearing in different movies, for instance of use in recommendation systems, *e.g*., Netflix. We apply our measures to the Marvel Cinematic Universe (MCU), and show what they teach us about this highly successful franchise of movies. In particular, these measures provide a useful predictor of success for films in the MCU, as well as a natural means to understand the relationships between the stories in the overall film arc.

## Introduction

The Marvel Cinematic Universe (MCU) is arguably the most successful movie franchise of all time. It has grossed more revenue from the films alone than the next two most successful (Harry Potter and Star Wars).

What makes the MCU so successful? Clearly, there are many factors: the deep and expansive nature of the source material they draw on, the clever adaptation to movie format, the talented actors and directors, and the number of pre-existing fans. However, there are other similar attempts to translate graphic novels to the big screen, none as large or successful as the MCU.

The cast of a movie has a very important impact on its success. Most obviously, “star power” can attract an audience, and ultimately the talent of the actors and director are critical. However, underlying this is a question of just how big the cast is. Details often make a character, and so smaller cast of more carefully constructed characters might be better; or perhaps volume carries weight? We need a way to measure cast size in order to consider which of these hypotheses is more true.

On a superficial level, it seems trivial to count the number of characters in a movie—just read the credits. But that is naive. Movie credits are not a uniformly defined source of data: the onscreen credits of *Iron Man 3* list 110 characters whereas for *Thor: Ragnarok* they list 43. These numbers hide the fact that the number of meaningfully contributing characters in these movies is similar.

Further, not all characters in a movie are equal. Most obviously, there are named and unnamed characters. The first are those who have the importance to require a proper noun designation, the second require a designation but are not important enough to warrant a real name and can include characters without lines such as those in the background of a scene. Counting these so-called extras is problematic because many of them are unlikely to be mentioned by name in the credits. And more importantly, should a profusion of extras carry the same weight in a metric of cast size as the number of named characters? *Ben-Hur* (1959) won a reputation as an epic in part because of its reputed 10,000 extras, but should these each count as much as its star Charlton Heston?

We argue in this paper that an *effective* measure of the cast size is useful, and such theoretically appealing metrics can be derived from those used to measure ecological diversity [1–3]. Such a metric could be useful

- in creating better predictors for movie success;
- in taxonomic classification of movies for subsequent analysis; and
- as a feature for recommendation systems.

The metric we focus on here is a effectualisation of Shannon entropy. However, entropy (and other metrics) are derived from a probability distribution. The second major platform of this paper is that the probability distribution underlying the metric is of great interest: we investigate two here, one based on dialogue and the other on the relative participation in *conflicts* within the film.

We will show that the Shannon-entropy effective cast-size metric can measure the size of a cast in a way such

- that it have an intuitive meaning (*e.g*., meaningful units);
- that it be built on firm mathematical foundations;
- that it be practically measurable, and that it be insensitive to noise in the data collection process;
- that it be hard for a studio to *game*, *i.e*., it be hard to artificially alter significantly; and
- that it is *effective*, *i.e*., numerically comparable across movies, and in particular useful for prediction or classification tasks at least in the MCU.

One of the surprising results of the analysis is that when entropy-based distances are used to create a projected plot of the movies they seem to naturally separate into a spectrum that categorises the major heroes in terms of personality types. We might see one axis of the plot as showing a transition from *thinking* to *feeling*. For instance, we see characters such as Iron Man/Tony Stark and Captain America/Steve Rogers towards the left: both make structured (if different) decisions based on codified, consistent sets of rules. Characters such as Star-Lord/Peter Quill from the Guardians and Thor who often act intuitively or emotionally (sometimes illogically from the perspective of other characters) are plotted towards the right of the chart.

Of course, any single metric will not capture all of the richness and detail of a movie franchise, and this technique is proposed to be used as part of larger suite of analysis tools. Moreover, as the focus here is on the MCU, we cannot necessarily generalise all results in this paper to the wider screen universe. However, by studying this narrow cross-section of the movie world, we can understand aspects of the metrics being used that might be obscured in a larger more heterogeneous dataset.

## Related work

Quantitative analysis of stories begins in most cases with *content analysis* of the text of the story itself. Studies can be broadly categorised into two main approaches: *Temporal* studies tend to focus on aspects internal to the progression of the story, such as the ‘arc’ of the narrative, while *structural* studies focus on the relationships between characters, often through social network analysis. The present work fits most closely with the *structural* approach as it focuses on measuring the effective number of characters, however we borrow some techniques from information theory that are most typically associated with the *temporal* approaches.

*Temporal analysis of stories*—While analysing plot structure has an extremely long history, arguably dating back to Aristotle (the Poetics), in modern (Hollywood) filmmaking the notion of the “three act structure” characterised by rising tension followed by resolution is codified/popularised by Syd Field [4]. Taxonomies of stories more generally have been debated throughout the 20th century, with characteristic numbers of plots/stories found by authors apparently decreasing over time, from thirty-six in 1916 [5], to twenty in 1993 [6], to seven by 2004 [7]. Entropy has been extensively used in analysis of language, *e.g*., see [8]. Indeed, Shannon’s original paper introducing information theory discusses redundancy in Joyce’s “Finnegan’s Wake”, and later non-parametric entropy estimators have been employed to quantify entropy in literature [9], plays and poems [10], and online social media [11]. Perhaps most similar to the methodological approach in this work are the studies on vocabulary [12] and on placing information-theoretic bounds on human language acquisition [13], where entropy is used as a measure of language capacity. Such approaches use information-theoretic techniques to estimate an “effective” vocabulary size, in a similar manner to how we estimate the effective cast size.

At a more fine-grained level of temporal analysis over the course of a story, Vonnegut proposed studying the “shapes of stories” by charting “ill fortune-great fortune” along a “beginning-end” axis [14]. This notion was made quantitative by analysing story “arcs” (which we distinguish from “plots” here) by applying sentiment analysis techniques to the text of written works. Notable examples here include the body of work by Jockers and coauthors [15–18], as well as Reagan *et al*., who find six characteristic emotional arcs in novels [19]. For films, there is some suggestion that these emotional arcs relate to the eventual success of the film, as measured through box office returns [20]. In this paper we find relationships between statistics of MCU films and box office returns as well, but through our analysis of effective cast size.

*Structural analysis of stories*—The present work is more closely related to the literature on analysing structural properties of stories using networks, either between characters or portions of text. Authors have studied the complex networks encoded in stories from throughout history, from ancient myths and sagas [21, 22], to Romantic novels [23, 24], to films [25]. Of particular relevance here are recent social network analyses of the Marvel (comic) social network [26] and some social networks from superhero movies including *Wonder Woman* and *Thor* [27]. Edwards *et al*. [28] compare different ways of constructing social networks based on scripts, focusing on the television series *Friends*.

To some extent this paper unifies the two major approaches to the quantitative analysis of stories by using entropy, which is typically associated with temporal analysis, to quantify a structural feature of the films we study, namely the effective cast size.

## Materials and methods

### The anatomy of a cast list

We are interested here in the cast of a movie, its *dramatis personæ* so to speak, but we are interested in the story, not its means of implementation, so we are interested in the number of characters (or parts or roles), not the number of actors (the two are not identically the same). The most obvious place to access the list of characters is through the *credits*. A movie is constructed by a long list of creative artists, actors and technical experts. Their contribution to the movie is generally acknowledged through these credits, which are displayed on screen at the beginning of a movie in the title sequence and near the end in the end credits. The credits are incredibly important to today’s movie participants because they effectively form their curriculum vitae.

However, there is no unique definition of how a credit list is constructed, and it has varied historically and regionally in level of detail and the types of activity that are credited. In the United States, the standardisation of credits starts around the 70s. However, standards continue to evolve: *e.g*., since *Toy Story* (1995), lists of “Production Babies” born to crew members have been increasingly included in movie credits. Superhero movies have, since *Superman* (1978), which had the then longest credit list at its release, often had very long lists, now sometimes including thousands of participants.

In the context of this paper the credits are important because they include a cast list. However, credited cast lists are often incomplete and sometimes misleading. There are many reason for this, and discussion requires some understanding of the different types of roles within a cast. Definitions for such vary (often base on local union specifications) but loosely we speak of

- Main roles: these are the primary roles in a movie. There are usually only a small number of such roles, and these characters perform the majority of action and dialogue.
- Bit parts: these are smaller roles, often defined by the number of lines of dialogue the character has (less than 6 lines seems a common standard).
- Extras: (or background talent, or atmosphere, or supernumeraries) these are much smaller roles, often as simple as providing background in a scene. The number of extras varies tremendously, potentially into the thousands. Extras are usually “silent” roles, though in the UK they are allowed to speak up to 12 words.
- Cameos: these are small roles, usually played by well-known actors or personalities. Stan Lee performed one of the most famous series of cameos. Historically, he appeared in almost all Marvel movies (exceptions are the Punisher movies and *Elektra*). He even appeared posthumously in *Captain Marvel* and *Avengers: Endgame*. Most recently, however, there was no cameo appearance in *Spider-Man: Far From Home* or *Dark Phoenix* and so we have presumably reached the end of this era.
- Doubles: stunt, body or costume doubles for a main actor fill the same character role with a different actor for various reasons.

Credit lists focus on the more important roles. Extras, cameos and doubles may be omitted for various reasons [29, 30]. Actors may not want to be listed for artistic reasons (to enhance anonymity of a character where that is important) or personal reasons, and movie producers may not want spoilers to be disclosed through a cast list (these days cast lists are often available before the movie release). Producers may also not wish to credit actors because this may have implications for employment costs, or simply because it dilutes the meaning of being given credit. There are also many new types of participation modes for actors (*e.g*., voice only, or motion capture), and movies appear in multiple formats (with different cutting) and it is ambiguous how these might be credited, *i.e*., should a character who only appears in a deleted scene be credited?

IMDb lists credited cast, but also adds to cast lists for movies, using data provided by fans identifying actors on screen, or actors self-identifying. Although such contributed information can have inaccuracies, it allows a window into how many roles are credited or uncredited [29, 30]. IMDb also lists additional attributes for roles: *uncredited, voice, motion capture, archive footage, scenes deleted, or credit only* [31]. Thus the data is valuable, but it still has many issues. Most notably, consistency is not enforced between movies, so a character (or even an actor) might be listed under different names (see below for more discussion) or given credit or not for similar roles in different movies. And the number of uncredited roles can vary tremendously.

Thus although the above categorisation is useful in some respects, it is imprecise. We therefore consider a second categorisation of characters that is more precisely defined in order to provide a fairer benchmark against which to compare entropic measures.

We divide the characters in a cast list into subclasses based the notion of “naming”, *i.e*., we break the characters into the classes:

- *Named*: These are characters that are assigned a proper noun designation, e.g., “Iron Man”.
- *Unnamed*: These are characters that are only indirectly names, *e.g*., “Tony Stark’s secretary”, or given a job name or other designation, e.g., “SHIELD agent”.
- *Minor roles*: These are characters that only appear as “uncredited” in lists such as that of IMDb, or are not noted on any list. Even as such, they may be significant, *e.g*., as the victim of another character’s actions.

The list of named characters has a more consistent meaning than the credited characters, but we will show that such count-based measures still have problems for assessing cast size. Our primary data will be informed by the *content* of movies, explicitly to avoid such issues.

### The movies of the MCU

The MCU includes TV series, one-shot short movies and other media, but here we regard only the canonical series of movies (released as of May 2019) as listed in Table 1.

**Table 1:** Facts and figures for the MCU movies. The ordering is largely alphabetical, but modified to group related movies. Box office (US domestic) and production budget are measured in millions of $. Speaking parts refer to the number of roles that speak at least one line, as measured from the available scripts.

Title	Type	Credited	Named	Speaking	Budget	Box Office
Ant-Man	origin	67	30	32	130.0	180.2
Ant-Man and the Wasp	sequel	61	27	10	130.0	216.6
The Avengers	team-up	53	23	49	225.0	623.3
Avengers: Age of Ultron	team-up	67	34	36	330.6	459.0
Avengers: Infinity War	team up	56	42	46	300.0	678.8
Black Panther	origin	66	22	38	200.0	700.1
Captain America: Civil War	team-up	103	25	41	250.0	408.1
Captain America: The First Avenger	origin	95	25	64	140.0	176.7
Captain America: The Winter Soldier	sequel	73	27	49	170.0	259.7
Captain Marvel	origin	51	30	39	175.0	425.4
Doctor Strange	origin	31	22	19	165.0	232.6
Guardians of the Galaxy	team-up	72	26	35	170.0	333.2
Guardians of the Galaxy Vol. 2	sequel	48	34	34	200.0	389.8
The Incredible Hulk	origin	72	16	10	137.5	134.8
Iron Man	origin	63	26	11	186.0	318.6
Iron Man 2	sequel	61	30	41	170.0	312.4
Iron Man 3	sequel	110	39	27	200.0	409.0
Spider-Man: Homecoming	origin	64	37	77	175.0	334.2
Thor	origin	50	29	42	150.0	181.0
Thor: The Dark World	sequel	39	28	38	150.0	206.4
Thor: Ragnarok	sequel	43	18	33	180.0	315.1

In order to better understand results, the movies are classified into types within the MCU based on the their characteristics and relationship to other movies in the franchise. We classify movies as

- *origin* movies—these are the first major appearance of a character, and often explain some of that character’s back story;
- *sequels*—these are movies that follow on directly from another with a large overlap in cast and plot elements; and
- *team-ups*—these movies involve a group of superheroes forming into team for a larger purpose.

The classification is soft in the sense that many of the movies have aspects of more than one type of movie: for instance, most movies in the MCU are sequels in some sense. Other movies such as *Guardians of the Galaxy* may be both a team-up and an origin movie (for the characters in the team). Here we identify the primary role of the movie. The classification is given in Table 1.

### How not to measure cast size

In ecological terms a “richness” metric just counts numbers of species. Here it would be a direct count of the characters in a movie. In ecological settings this is well-known to be naive [3]. In this section we demonstrate that such counts are naive here also.

The numbers of credited and named characters for each movie are given in Table 1. There are many notable defects that one observes, for instance with regard to the number of credited characters:

- *Iron Man 3* apparently has the largest cast. The movie, however, is a relatively straightforward linear plot with the lead character (Iron Man/Tony Stark) being dominant in terms of scene time and action.
- *Avengers: Infinity War*, which has a cast that includes almost every prior superhero from the franchise (a notable exception is Hawkeye/Clint Barton) has a rather intermediate credited cast.
- *Thor: The Dark World* also has a rather small list cast, given it takes place over three settings (Asgard and Earth and the eponymous Dark World) with three corresponding sets of cast members.

There are also issues with regard to the number of named characters:

- *Thor: Ragnarok* is listed as having the second smallest set of named characters. As with the other Thor movies this takes place over several settings, each with its own cast, and it involves alliance between Thor (and his usual team) and the Hulk/Bruce Banner and other new characters.
- *Spider-Man: Homecoming* is listed as having one of the largest sets of named characters. In this case, there are indeed quite a few such, but they play very little if any role in the plot.

However, a more important problem with this metric is it provides little separation between movies. The majority of movies all lie in a narrow band in the middle of the distribution. This lack of differentiation means the metric is not terribly useful.

We can study the question of separation more quantitatively. Two indexes (the Dunn index [32] and the Davies–Bouldin index [33]) of the separation between types of movies are given in Table 2 for the various measures of cast size considered here. Note that larger values for the Dunn index indicate better separation, but smaller is better for the Davies-Bouldin index. They are both scaled such that the measure is unitless, and hence the varying ranges of the cast-size estimates do not matter.

**Table 2:** Separation indices for the type of movie derived from the four cast-size metrics. Note that larger is better for the Dunn index, and smaller is better for the Davies-Bouldin index. The best estimate (emboldened for ease of reading), by a large margin, is the Shannon metric.

Metric	Separation index	
	Dunn	Davies-Bouldin
Credited	0.028	48.90
Speaking	0.049	24.11
Named	0.087	15.88
Shannon metric	**0.257**	**3.91**

The very noticeable result is that separation is similar between the count-based metrics: credit, named and speaking characters, with named being best amongst these three. The irony is that determining named characters is the most manually intensive approach as it requires discrimination between titles such as “Tony Stark” and “Tony Stark’s secretary.”

The separation indexes are overwhelmingly better for the Shannon metric, which we shall describe below.

The implication is that naive estimates of the number of characters in a movie perform badly in at least one task, separating types of movies. The reason lies in the examples given above. There are many exceptional cases where one or the other of the movies has unexpected numbers (either larger or smaller) of characters as measured by the count-based metrics.

A final issue with simplistic count-based metrics is that they are easily *gameable* in the sense that it would be easy for a studio to artificially alter the metric on a given movie by manipulating the number of minor roles (in credits or speaking minor lines). If such a metric became an important index to describe movies, then studios might attempt to game it for financial or other reasons. A metric that is harder to game is therefore more desirable.

The Shannon metric is described below in terms of character participation which we define next.

### Measuring a character’s participation

We would like to measure each character’s contribution to a movie. However, this is difficult. Obvious measures such as screen time are difficult to obtain. More importantly, screen time doesn’t necessarily take account of whether a character contributes meaningfully during this time. For instance, a scene with a large group of extras is giving screen time to those extras, but their contributions may be minor, leading to substantial over-estimates of the cast-size metric.

Investigation of screen time deserves more consideration, when and if a means to measure it becomes evident. However, there are alternative proxy measures of each actor’s scale of contribution. Many movies are driven by dialogue: what the characters say informs us of the plot and the attitudes and emotions of the characters. Other aspects are important, their actions, the setting, the background music and so on, but the dialogue is the platform around which these aspects revolve. Hence, one approach to measuring the participation of a character in the movie is to measure the number of lines of dialogue they speak.

This is not the only metric, or even uniquely the best. Some characters may be laconic, or overly verbose. And the importance of any given line may vary. At a deeper level, however, not all movies are driven by dialogue. A musical or opera is driven by the music (the dialogue and plot are often only there to provide connections between the songs). Pornographic movies are driven by sex, with plot and dialogue again providing (usually extremely minimal) connections. Here we are concerned primarily with the MCU, which falls into the specific superhero genre, and more generally into the action movie genre. And action movies are often driven by conflict. Thus measuring the number of conflicts in which a character participates will give us another measure of their level of participation in the movie.

### Effective population size measurement

Our goal is to find a more effective metric for the “population size” of the cast of a movie. There are several goals in forming such a metric, described in the introduction. Luckily the problem has been considered extensively in the context of measuring ecological diversity of a habitat (for a review see [3]), and there are several parallels. In ecology there is a need to understand not just how many members of each species are present in a habitat, but to capture what this means in terms of diversity. The problem is complicated by the noise in estimating species numbers, and the underlying variation in those numbers. For instance, a naive count of the number of different species can be misleading if most of those species are near extinction.

However, there are several ecological metrics, and their purpose is different, so rather than choosing one without care, we will consider here the goals of such a metric in *axiomatic* terms [34]. That is, we shall describe (mathematically) the properties that such a metric should have in our specific context, and thereby derive a suitable metric.

First, such a metric should be based on the proportion of contribution of each actor, not other features (such as their name). Hence, we can write the effective number as a function of the proportional contributions of each character to the story, *i.e*., if we write the effective number as $\hat{N}$ then it is a function $\hat{N}\left(p_{1},p_{2},\ldots ,p_{m}\right)=\hat{N}\left(p\right)$ where the proportional contribution of character *i* is *p*_*i*_ and there are *m* characters to consider. Given this definition we have the axioms.

- **Non-negativity**: $\hat{N}\left(\cdot \right)\geq 0$. It makes little sense to report a negative population size.
- **Continuity**: $\hat{N}\left(\cdot \right)$ is a continuous function of the variables: small changes in the data should result in small changes in the metric. This is important for robustness, but also in avoiding gameability. In order to change the metric a studio should have to make significant changes to the content, not artificially manipulate minor aspects of the movie.
- **Symmetry**: Reordering the actors, for instance such that *p*_*i*_ and *p*_*j*_ are swapped, should not change the metric. For instance
  $$\begin{array}{c} \hat{N}\left(p_{1},p_{2}\right)=\hat{N}\left(p_{2},p_{1}\right). \end{array}$$
  This condition arises because we want a metric in which characters are interchangeable in order to be able to make comparisons, for instance, between different movies.
- **Normalisation**: If there are *M* equally contributing characters, *i.e*., *p*_1_ = *p*_2_ = ⋯ = *p*_*m*_ = 1/*M*, then we require that $\hat{N}=M$, and this should be maximal. This condition is imposed to scale the values intuitively, so that a value of $\hat{N}$ can be associated to an actual number of characters.
- **Zeros**: Adding a character that makes no contribution should not change the metric, *e.g*.,
  $$\begin{array}{c} \hat{N}\left(p_{1},p_{2},0\right)=\hat{N}\left(p_{1},p_{2}\right). \end{array}$$
  A net result of this and the prior condition is that $\hat{N}\left(1,0\right)=1$. This condition arises both for technical reasons, and because we don’t want noise (small errors) to lead to instability in the metric. For instance, introducing a character who makes very little contribution should not change the metric by much.
- **Monotonicity**: If the number of participating characters increases, then the metric should increase, *e.g*., assuming all participation is non-zero
  $$\begin{array}{c} \hat{N}\left(p_{1},p_{2}\right)<\hat{N}\left(p_{1},p_{2}^{\prime },p_{3}^{\prime }\right), \end{array}$$
  where $p_{2}^{\prime }+p_{3}^{\prime }=p_{2}$ and $p_{2}^{\prime },p_{3}^{\prime }>0$. The intention is that larger casts should report a larger metric.

Additionally we expect the metric to be sub-additive in the sense that if one considers two movies jointly, then the effective cast size of the joint movie should be no larger than the sum of the casts of the two components.

This list of axioms has redundancies, but more importantly it does not lead to a unique metric. There are several alternatives that are valid. We use the method based on Shannon entropy *H*(**p**) [34], which is defined as follows:

$$\begin{array}{ccc} H(p) & = & -\sum_{i}p_{i}\;{\text{log}}_{2}\;p_{i}, \end{array}$$ (1)

$$\begin{array}{ccc} \hat{N}\left(p\right) & = & 2^{H(p)}, \end{array}$$ (2)

with the common convention that 0 log 0 = 0. The metric $\hat{N}\left(p\right)$ has sometimes been called the *perplexity* of a distribution (because of its relationship to one’s ability to predict the outcome of a random event with this distribution) and has been used in natural language processing [8]. The use of this metric in ecology was pioneered by Margalef [1], but the method and its utility were perhaps better explained by later proponents of the ideas such as MacArthur [2], who used it, for instance to show how lizard species diversity in 9 desert regions increased approximately linearly with the volume of scrub available as habitat.

These axioms roughly correspond to axioms used to derive Shannon entropy, but there is a key grouping/partitioning/recursivity axiom missing here as it appears hard to justify in the context. Hence there are other metrics that satisfy these axioms [34], but the metric we have chosen has several advantages: (i) Shannon entropy is easy to calculate, (ii) it is used in many other fields (*e.g*., physics and information sciences) and so has common interpretations, (iii) the maximum entropy principle naturally leads to models, and (iv) entropy has many generalisations, for instance to distance metrics—this is a key advantage here in that we can adapt the same ideas for comparisons between movies.

### Estimating H

The metric leaves open the means by which we estimate the *p*_*i*_, the proportional contributions of each character. We test the use of dialogue or conflict as explained in later sections. However, in movies (as in ecology) it is rare for us to know the exact proportion or actors’ participation (or species abundance). Thus we view the values *p*_*i*_ as estimates which we denote ${\hat{p}}_{i}$ from which we estimate entropy. The most obvious estimator using direct application of (1) results in a consistent, asymptotically normal estimate [35], that is, the result would converge to the correct value given a large amount of data with the errors well approximated by a normal distribution. However, for finite data the estimator is *biased*. We instead use Basharin’s estimator [35],

$$\begin{array}{c} \hat{H}=-\sum_{i=1}^{m}{\hat{p}}_{i}\;{\text{log}}_{2}\;{\hat{p}}_{i}+\frac{m-1}{K}\;{\text{log}}_{2}\;e, \end{array}$$

where *m* is the number of categories in the distribution, and *K* the total number of samples. Basharin’s estimator corrects the first order bias. The result is that the estimate will on average be closer to the true value for small *K*.

It also has the advantage that we know a first order approximation to its variance:

$$\begin{array}{c} \text{Var}[\hat{H}]=\frac{1}{K}[\sum_{i=1}^{m}p_{i}({\text{log}}_{2}p_{i}{)}^{2}-H^{2}]+O(\frac{1}{K^{2}}). \end{array}$$ (3)

We estimate this variance using ${\hat{p}}_{i}$ and $\hat{H}$ and use asymptotic normality to construct Gaussian confidence intervals for our estimates of entropy. We then transform these when calculating $\hat{N}$ to obtain intervals for this estimate as well.

## Data

All collected data complies with terms and conditions of the specific data sources with details of individual datasets provided below. Data, where allowed by licensing, is hosted on GitHub [36].

### Metadata

The majority of movie metadata (e.g., duration, production budget, box office revenue, etc.) were provided by “The Numbers [37] powered by OpusData [38]” under an academic license agreement. The data was downloaded as of the 25th of May, 2019. The data thus excludes the most recent Marvel movies, and some revenue figures may not be completely up to date, though we focus on box-office revenue in order that this not cause significant errors in the analysis.

Cast lists were accessed through the third party, open-source IMDbPy API [39] to IMDb. The advantage of this listing is that it includes many uncredited roles that have been added and verified through IMDb’s interactions with the production community.

### Scripts

Publicly available movie scripts were used as a source for dialogue. This has been a valuable data source in previous work [40], but has been focused in constructing dialogues for use in computational linguistics [41]. Here we are not analysing the text of the speech, but rather the speakers.

No single source has scripts for all of the MCU movies. We used two sources:

- The community run Transcripts Wiki on the website *fandom.com* [42]. These webpages were downloaded and processed to find lines of dialogue and their speakers. The scripts come in a number of formats, each requiring different methods of parsing. Some scripts had inconsistent formats or were incomplete (see below for more discussion).
- PDF documents of movie scripts were sourced from Script Slug [43] if they were unavailable or incomplete on the Transcripts Wiki. The text from the PDFs was extracted and parsed to collect the names of each speaker of dialogue. Individual care was put into parsing each PDF as formats varied.

Transcripts, especially fan transcripts, often lack the depth of information desired for more complex analysis [44], but the extraction of speakers in dialogue sequences is relatively reliable. However, as noted, incompleteness was a problem.

Even with two data sources we did not have complete scripts for all movies. Only 14 movies have complete scripts. Three have mostly complete scripts—denoted *partial*—that may miss a scene or have minor uncorrected issues. The four remaining *incomplete* movies had large omissions. Details of the movies and numbers of lines of dialogue available in their transcription are shown in Table 3.

**Table 3:** Size and completeness of data for each movie including the numbers of transcribed conflicts and lines of dialogue, and whether the dialogue transcription was complete, partially incomplete or largely incomplete.

Title	Run Time (m)	No. of conflicts	No. of lines	Status
Ant-Man	117	82	865	complete
Ant-Man and the Wasp	118	110	180	incomplete
The Avengers	143	237	830	complete
Avengers: Age of Ultron	141	302	975	complete
Avengers: Infinity War	149	278	991	complete
Black Panther	134	117	728	complete
Captain America: Civil War	147	263	982	complete
Captain America: The First Avenger	124	99	619	complete
Captain America: The Winter Soldier	136	149	822	complete
Captain Marvel	123	165	686	partial
Doctor Strange	115	112	159	incomplete
Guardians of the Galaxy	121	157	576	partial
Guardians of the Galaxy Vol. 2	136	127	956	complete
The Incredible Hulk	112	92	63	incomplete
Iron Man	126	81	124	incomplete
Iron Man 2	124	112	1006	complete
Iron Man 3	130	121	571	partial
Spider-Man: Homecoming	133	58	1558	complete
Thor	115	95	873	complete
Thor: The Dark World	112	106	732	complete
Thor: Ragnarok	130	197	970	complete

### Conflict transcription

Underlying this work is the desire to form a theory of conflict in narrative analysis. Much of narrative concerns conflict, for instance, between a hero and villain. The development of this component of a narrative is particularly obvious in the action movie genre, in which plots of movies are predominantly carried by a sequence of interdependent conflicts. Indeed, there are famous cases of movies whose main actor spoke almost unintelligibly, but carried the film through charisma and physical presence.

Mathematically, we can think of a story as having a set of characters C. A conflict is a mapping from a pair of these characters to an outcome, *i.e*., it is a function *f*_*i*_(*a*, *b*) → {*a*, *b*, *d*}, where a,b∈C and *d* means a draw or an indeterminate outcome. A scene is made up of a sequence of such conflicts *i* = 1, …, *n*. For instance, a movie scene often follows a backwards-forwards motif oscillating between minor victories for the villain and hero. Alternatively, the hero might be the underdog, physically over-matched by the villain, suffering repeated setbacks, but eventually, the hero wins by adopting a clever stratagem. We are not concerned in *this* paper with the temporal structure of this process. Here we are only interested in the proportionate contributions of each character to the action of the story, but future work aims to tease out such motifs from this data.

It is not always obvious from observation how to divide a story into a sequence of such mappings. Technical considerations aid in the decision: (i) if conflicts are pairwise, then we must divide conflicts involving more than two parties into components small enough to be modelled as pairwise, and (ii) estimates based on the data will be limited by the resolution of the data. Both of these considerations push towards as fine a division of conflicts as possible. On the other hand, there is a natural desire that the outcome *d* be in the minority, *i.e*., that most conflicts have a winner and loser, and this means that we cannot meaningfully break a scene into individual frames. Additionally, the overhead of a frame based approach is impractical for manual transcription, not to mention that individual frames are often meaningless without context. Thus we need to derive a list of conflicts that is fine-grained enough that we can observe the dynamics of a larger-scale sequence, but within the practical limits of transcription. A natural level might be at that of the *panel* in a graphic novel, but transcriptions of movies is less easy.

In order to make the transcription process more transparent, it is helpful to break the transcription process into a step-wise set of criteria:

1. We break a scene into pieces along natural junctures, *e.g*., pauses (for instance to engage in dialogue), changes of location, changes in the engaged pair of combatants, and knock downs or knock outs. Think of the divisions as the *gutters* in a graphic novel, *i.e*., the spaces between panels.
2. Each such sub-scene is classified into a type (typically physical conflict, but for example in movies such as *Doctor Strange* some conflicts are magical in nature).
3. The participants in the conflict are identified. Usually this is entirely obvious, but some participants may be unnamed characters, or may only be observed from behind or in some other way obscured. In these cases, identification may be through supplemental sources such as scripts, plot synopses and the like. In a small number of cases one character opposes two or more others simultaneously. In these cases we group the characters in the original data, but subdivide the groups when counting the number of conflicts.
4. The winner is determined. This is often obvious, however there are some subtleties. For instance, there are some cases where a stand-off, draw or other inconclusive result occurs. However, the winner will not affect the result of this paper.

Each conflict is given a time-stamp close to the start of the sub-scene division. We do not record duration because these are quite short, and the errors in such annotation would be relatively large.

Additional details such as factors that affected the outcome are included in the data, but are not germane to this study so we will not discuss them in detail here.

In order to make the process clearer we provide the following example: one of the most watched movies in the MCU is *The Avengers*. And one of the archetypal scenes in the movie is the meeting between Captain America/Steve Rogers, Iron Man/Tony Stark and Thor. In the meeting they each fight (over the captive Loki). The scene cuts quickly between pairs of characters fighting. It also incorporates a classic backwards, forwards dynamic between Thor and Iron Man where each wins for a short interval, and then the fight reverses. Overall, one might classify the fight as a draw, but the details are important. The transcription of the physical conflicts in the three-way fight between Iron Man, Thor and Captain America is given in Table 4.

**Table 4:** Three-way fight between Iron Man, Thor and Captain America in *The Avengers*, transcribed into a series of conflicts. Timestamps are given as minutes and seconds from the start of the movie (the absolute value of timestamps can vary depending on the movie format—in this case they are with reference to the version distributed on UHD disk). The ordering of Party A vs B is not informative. The transcription begins at the point when Iron Man attacks Thor, knocking him away from Loki, and ends when Thor strikes Captain America’s shield and the resulting shock-wave knocks Thor down (along with half the forest).

Timestamp	Party A	Party B	Winner
47.07	Iron Man	Thor	Iron Man
47.45	Iron Man	Thor	Thor
48.01	Iron Man	Thor	Iron Man
48.18	Iron Man	Thor	Thor
48.27	Iron Man	Thor	Iron Man
48.47	Iron Man	Thor	inconclusive
48.50	Iron Man	Thor	Thor
49.02	Iron Man	Thor	Thor
49.07	Iron Man	Thor	Iron Man
49.17	Iron Man	Thor	Thor
49.22	Iron Man	Thor	Iron Man
49.25	Iron Man	Thor	Iron Man
49.28	Captain America	Thor, Iron Man	Captain America
49.45	Iron Man	Thor	Thor
49.53	Captain America	Thor	Captain America

The loser of a conflict may contribute to the narrative at least as much as the winner so a character’s contribution to the movie is measured purely by the number of conflicts in which they are involved, not whether they win or lose.

We transcribed the conflict data manually by viewing and annotating the movies. During transcription the movie was paused and rewound (frequently) so that notations could be made immediately, avoiding problems of memory. In transcribing we are aiming to balance the alternative pressures: to subdivide scenes at a fine granularity vs the desire for conflicts to have determinate outcomes. There is inherent subjectivity in any such transcription process. How finely to subdivide conflicts, and even who is victorious is often straight-forward, but not always. Ideally we would involve multiple transcribers, and use a metric of inter-coder reliability to determine a consistent set of data. However, (i) the movies represent a large corpus of material; (ii) conflict data does not fall into a standard model for inter-coder concordance (commonly used correlation coefficients are designed to assess agreements amongst ratings, not between choice of subdivision of a dataset), and (iii) we acknowledge that the theory of conflicts described so far is not fully formed at present. Instead, we aimed here to at least provide a dataset that was consistently transcribed, and to build a metric of effective cast size that was robust to transcription variations.

In order to reduce inconsistency, all movies were examined and transcribed by a single observer. In addition, the order of transcription was randomised (constrained by movie releases) to avoid systematic biases. Transcription was performed over as short a period as practical for the same reason. The main body of the MCU was transcribed over a few weeks, but transcription of *Captain Marvel* occurred somewhat later when it was released outside theatres. *Avengers: End Game* and *Spider-Man: Far From Home* were not included because they were not released at the time of the study.

A more difficult issue to address is that as transcription progressed, there was a parallel learning process about how to most accurately and consistently transcribe events. In particular, the level of subdivision, and the degree of inclusion of conflicts between minor parties increased in later transcriptions as their importance was realised. In order to address this drift in transcription we reprocessed the first three movies. The second transcription of these movies was substantially different from the first, which contains a much courser breakdown, and hence many fewer conflicts. This data allows for a comparison to test the sensitivity of our metric to the collection methodology (see below).

In general, releases of movies in different regions can be cut slightly differently, for instance to fit into a particular ratings scheme. The movies transcribed were all the standard Australian release. We did not analyse “director’s cuts” or other such modified releases. We have not seen any evidence that the MCU movies released in Australia differed from the US release, or any other worldwide releases.

### Data cleaning

It’s often said that 80% of the effort in a data analysis is spent on data cleaning, the process of getting the data ready to analyse.

Hadley Wickham [45]

The conflict data described above was collected as expeditiously as possible. It was quickly typed into a spreadsheet, resulting in typographic errors. Likewise fan transcripts contained some errors. These errors were detected by parsing the data, and validating against a set of criteria, *e.g*., time sequence monotonicity, name frequency, and simple matching algorithms; as well as matching to external data sources. Errors were corrected manually, with reference to the original movie where the mistake was not obvious (typos were generally very obvious once discovered).

A more critical issue lies in character names. Many characters appear in multiple MCU movies. However superhero names are not standardised. Characters introduced in one film under a given name are sometimes reintroduced under another (*e.g*., Bucky Barnes vs the Winter Soldier). Also naming conventions in movie credits are not standardised, and superhero movies add a layer of complexity because superheroes often have aliases. For instance Natasha Romanoff is known as the superhero Black Widow, but also appears in credits as “Agent Romanoff” and “Natalie Rushman”, and abbreviations or combinations of these. Characters also sometimes appear in alternative forms, *e.g*., “Young Gamora,” or are known by nicknames such as “Cap”. The dialogue in scripts also uses variant forms of these names. An aliases file was created in order to disambiguate characters and convert all to one name. Extensive use was made of authoritative sources of data on the MCU—in particular [46]—in order to ensure accuracy of the alias lists. Nearly 200 characters are listed in this data, commonly with two aliases, but sometimes with significantly more. For example, The Winter Soldier, James Buchanan ‘Bucky’ Barnes has six.

Unnamed characters do not use the alias list, and some generic characters may be grouped together by a credits lists or the names used in scripts. For instance, two separate characters may be listed as “SHIELD Agent”. The number of such, and the number of conflicts and/or lines of dialogue that such characters appear in is small, but may result in a marginal decrease in the effective cast size. A key advantage of the approach proposed here is a high degree of robustness to such noise.

Data, where allowed by licensing, is hosted on GitHub [36]. Apart from our analysis, it includes summaries of metadata, aliases, conflict lists, and raw scripts where allowed (FANDOM’s terms of use https://www.fandom.com/terms-of-use grant use of the material under the Creative Commons, Attribution-ShareAlike 3.0 Unported (CC BY-SA 3.0) license, however Script Slug’s terms of service https://www.scriptslug.com/legal only grants the right to temporarily download one copy of materials for non-commercial transitory viewing).

## Results

### Effective (conflict) cast size

**Fig 1:**
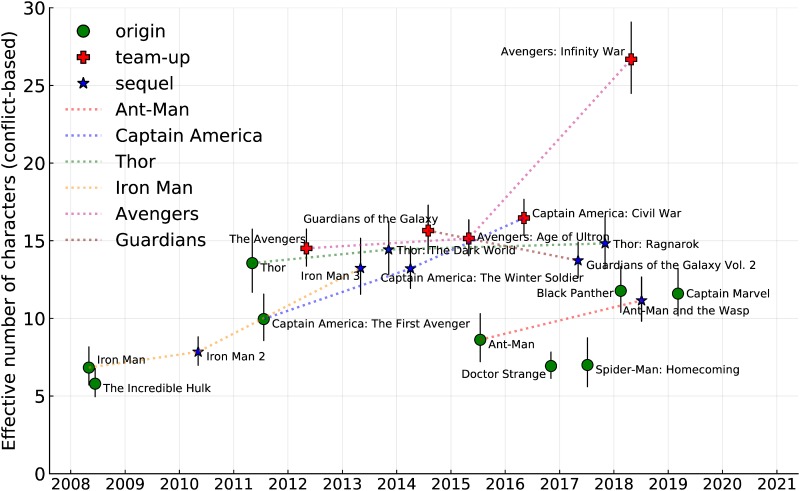
Effective conflict-based cast size of each movie in the MCU showing type of movies by shape, and sub-sequences connected by dashed lines. The *x*-axis is the theatrical release date. Equivalent figures for named and credited characters are included in the supporting materials.

The movies considered are listed in Table 1. We consider first the effective cast sizes obtained from the conflict data because of the completeness of this data. Effective cast sizes are shown in Fig 1 with the type of movie indicated by the marker, and specific sub-sequences of the overall set of movies are indicated by dashed lines. There are many interesting features of this plot.

When considered by class we see notable features: most origin movies have a small effective cast, which grows in sequels. The degree of inflation is expressed quantitatively in Table 5. For instance, the two sequels to *Thor* have increases in (conflict-based) effective cast size of 6.4 and 9.3% respectively. The averages show a small increase for first sequels, and a much larger increase for second sequels, but interpretation of this result should be tempered by the small number of second sequels being considered.

**Table 5:** Inflation of conflict-based effective cast size in sequels. The results are given as percentage increases with respect to the initial movie in a sequence. For instance, the two sequels to *Thor* have increases in effective cast size of 6.4 and 9.3% respectively. The only decrease recorded is for the second Guardians of the Galaxy movie.

Sequence	Inflation (%)	
	1st Sequel	2nd Sequel
Ant-Man	29.4	-
The Avengers	4.4	83.9
Captain America: The First Avenger	32.7	65.3
Guardians of the Galaxy	-12.3	-
Iron Man	14.8	93.8
Thor	6.4	9.3
Average	12.6	63.1

The exception to inflation in sequels is the Guardians’ sequence of movies. Thor’s sequence also has a more stable cast size (though the actual cast, and even the distribution varies). The movie *Thor* is unusual as an origin movie because it takes place across two major settings (Earth and Asgard) each with their own somewhat disjoint casts. It is therefore not surprising that the effective cast size is approximately double that of other origin movies. The Guardians of the Galaxy movies start with a team-up movie without the typical origin movies for team members, making these movies an outlier within the MCU, but leading to consistent casts sizes when considered by type.

**Fig 2:**
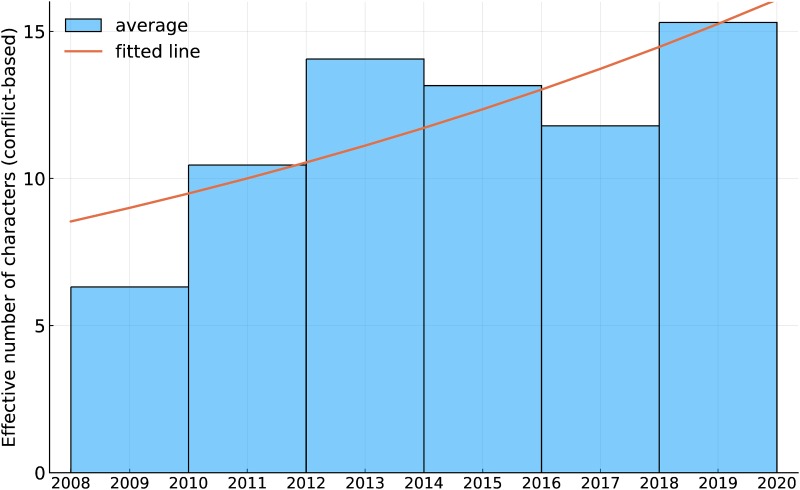
Effective conflict-based cast size for each two-year block since 2008 (bars) and a fitted exponential curve.

Another notable feature is a general level of overall inflation in the cast sizes with time. This is quantified in Fig 2, which shows the average conflict-based cast size in two year blocks (the choice was a compromise between seeing time-detail and having sufficient movies in each block to establish an average), and an exponential fit (corresponding to a constant inflation rate) to the data. The model fits with *p*-values 0.0225 and 0.0195 for the coefficients indicating that at *α* = 0.05 the fit is significant. The exponential rate of increase corresponds to a yearly increase in cast size of 5.4%.

These results appear to fit well with intuition about the structure and evolution of the MCU. Many fans or movie critics might have drawn plots with a similar shapes given a little thought, but deriving this from actual cast lists is hard, as we showed earlier (and in the supporting material). It is a measure of the success of the metric that it should match with intuition about the movies.

We can also consider the overall cast size of the MCU. If we combine the movies by taking averaged proportional contributions from the full set of characters, then the effective cast size is approximately 119.6 characters. This is an extraordinarily large number for a franchise, and reflects both Marvel’s willingness to develop multiple streams of movies building up to the central *Avengers* movies, and the fact that the action is well-distributed amongst this cast, *i.e*., there is no single, central character that dominates the movies. Compare, for instance, to the James Bond franchise which has a single, extremely dominant character, the eponymous James Bond.

On that topic, the top-5 characters that make the largest contributions to the overall metrics are in order:

1. Iron Man / Tony Stark;
2. Captain America / Steve Rogers;
3. Thor Odinson;
4. The Hulk / Bruce Banner; and
5. Black Widow / Natasha Romanoff.

Again, there is no surprise here, but the relatively equal contributions from the top-5 characters is somewhat unique in film franchises.

The large cast size of 119.6 characters is also interesting in comparison to a simple sum over the effective cast sizes of the movies (248.5). In ecological terms this disparity would be described in terms of *α*-diversity (the within habitat biodiversity) *β*-diversity (the between habitat biodiversity) and *γ*-diversity (the overall biodiversity of a set of habitats) [47]. The MCU sits in an interesting place where a significant part of its diversity occurs inside each movie (*α*), and a significant part between movies (*β*) but there is also a strong overlap (which we will consider further later in the paper).

### Movie success

I win my awards at the box office.

Cecil B. DeMille

A metric is as useful as its uses. In the context of movie production a key performance indicator is how profitable the movie is, *i.e*., the ratio of the movie’s box office receipts to its cost. There are many other facets to the success of a movie: for instance Star Wars is legendarily famous for its merchandising, but box-office receipts are (i) a somewhat easier number to obtain, and (ii) more immediate than other metrics, which is important in order to compare recent movies.

Some effort has gone into developing predictive models for movie performance, *e.g*., see [48]. Amongst many factors they consider (audience-based, release-based, and content-based) the content includes who is in the cast (that is the actor, not the character), and the actors’ popularity. They also note that features of the acting team can contribute, but again they are considering actors not cast, and they consider semantic features of the group (*e.g*., team chemistry) rather than purely quantitative measures. Instead, we test the effective cast size as a predictor: this is much simpler to measure as it is a feature of the movie itself, not the various qualities and relationships of the actors.

**Fig 3:**
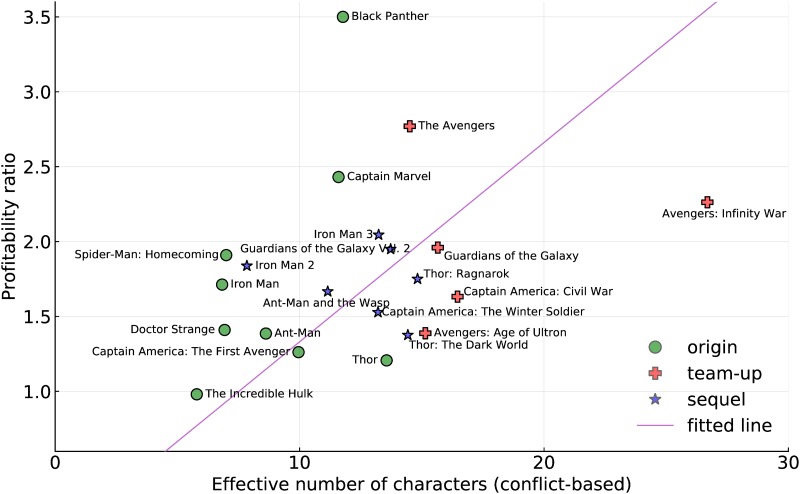
Profitability as a function of effective cast size (based on the conflict metric). The straight line shows a linear fit to the data (*p*-value < 10^−9^), illustrating the positive correlation between the metric and profitability.

In Fig 3 we compare the effective cast number to the profitability ratio. Of note in the MCU is that all but one movie have profitability ratio greater than 1, the exception being *The Incredible Hulk*, which was widely considered a failure (its lead actors was one of the very few replaced in later movies). However, its role in establishing one of the key characters of the franchise should not be underestimated. One of the clever ideas in the MCU seems to be the understanding that establishment movies would allow construction of larger, more interesting team-up movies later on.

We also plot on Fig 3 a least-squares fitted line through 0 (a movie with no cast would have profitability zero). The *p*-value for this fit is below 10^−9^ indicating strong significance to the relationship, and Pearson’s correlation coefficient is 0.184 indicating a non-negligible correlation between the two statistics.

There is considerable variation around the line, for instance there are quite a few movies that lie well above it: *Black Panther* stands well above the other movies in this respect. Quality of acting and direction, cast “star power”, timing and other factors cannot be discounted as important to the overall success of a movie. However, the effective cast size also appears to influence the profitability of a movie.

This is a fact that does not seem to be missed by the producers of the MCU franchise. As we noted earlier, larger casts seem to be becoming more common.

Why should this be? Do audiences really prefer a larger cast? We have to be very careful about this conclusion because the movies with larger casts tend to be team-ups. Dedicated fans might watch all of the MCU movies, but other fans may choose to watch only a single sub-sequence. When these culminate in a team-up, this draws in audiences from the multiple strands.

The fact that the MCU is constructed not of individual movies, or individual sequences is one of its chief beauties. Though two recent origin movies were very profitable in themselves, the producers seem to understand that smaller scale origin movies are a necessary pre-condition for the profit generating team-up movies.

**Fig 4:**
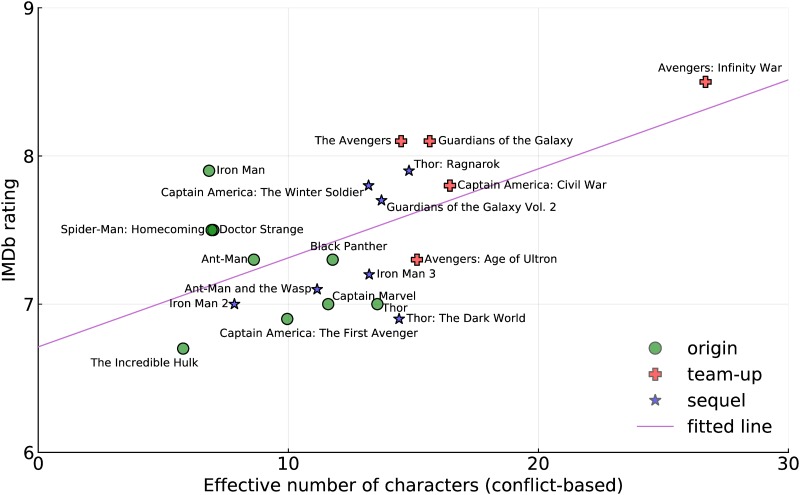
IMDb rating as a function of effective cast size (based on the conflict metric). Also plotted is a line fitted to the data (with *p*-values < 10^−15^ and 0.006).

We can also look at the relationship between ratings and cast size (see Fig 4) and we see the same type of pattern. The fitted line (with *p*-values < 10^−15^ and 0.006) shows the relationship between the two variables. Larger effective cast sizes are correlated with higher ratings, though once again we must note that correlation does not imply causation, and there is much variation. For instance, *Iron Man*, which was hailed as one of the best movies in the franchise and as one of the key initiators of the cinematic universe rates higher than this simple predictor would suggest. Many confounding factors are incorporated in this result, including the larger effective cast sizes for team-up movies, as indicated earlier, and the inflation in cast sizes that has been occurring over time, and so it would be simplistic to say that larger cast sizes lead to higher ratings. Never-the-less it seems an intriguing avenue for future investigation of other corpora.

### Robustness

A key concern in any such metric is the impact that noise in the underlying data might have on the results, particularly where there is an element of subjective judgement in the transcription process. To understand how sensitive the effective cast size is to transcription variations, we performed two separate transcriptions of three of the MCU movies. The two transcriptions were very different: the second set were performed at a much courser level. Table 6 shows the results. The number of conflicts found in each dataset is listed in order to show just how different these two transcriptions were.

**Table 6:** Conflict-based cast size estimates for three movies with two alternative views. Here *K* refers to the measured number of conflicts in the two transcriptions. The data includes 95th percentile Confidence Intervals (CIs) based on Basharin’s estimate (3). Note that the CIs for estimates from Datasets 1 and 2 overlap, suggesting that the difference is not significant.

Title	Dataset 1				Dataset 2			
	K	$\hat{N}$	± 95% CIs		K	$\hat{N}$	± 95% CIs	
Iron Man	81	6.83	5.72	8.16	36	5.01	4.04	6.21
The Avengers	237	14.51	13.36	15.75	92	14.23	12.64	16.02
Avengers: Age of Ultron	302	15.14	14.04	16.34	180	13.35	12.27	14.52

The effective cast sizes for each case are also reported. The notable feature of effective cast size is that it varies by a much smaller amount than the input data. There are some changes, mainly because in the courser grained analysis, some characters do not appear at all, and hence it makes sense that the effective cast size would decrease, but this is a comparatively small decrease as can be seen in comparison to Basharin’s estimate (3) of the 95th percentile confidence intervals, also shown in the table.

This robustness to input data variations is a key ingredient in any good metric.

### Gameability

Any metric can be distorted by artificially distorting its inputs. However, we desire a metric for which such deliberate manipulation—gaming—is hard. The Shannon-entropy metric was chosen from the start for its relative insensitivity to small changes in the inputs, and that results in the robustness of the metric to different transcriptions, as seen above. Here we consider the level of distortion of the metric that can be created by deliberately inserting additional minor roles into a movie.

We test gameability by inserting 5, and then 10 characters into the movie, each of which participates in 1 conflict. A movie studio might conduct such a manipulation purely through differential editing, *e.g*., by reducing the number of minor conflicts that are cut from the final version of the movie. Such editing would increase a simple count-based metric by the number of extra roles (5 and 10 in this case).

We performed the same addition on each of the movies in the MCU dataset and recalculated a gamed version of the Shannon metric. The result was average increases of 1.09 and 2.25 characters, respectively. This represents 21.8% and 22.5% increases compared to the number of characters injected. Thus, although the metric can be distorted, the amount of effort required to create a major distortion is large—the Shannon metric is about five times harder to game than count-based metrics.

### Dialogue-based effective cast size

So far we have only considered the conflict-based metric for cast size. In this section we consider the related dialogue metric—as a reminder this uses the same formulation but an alternative estimate of the proportional contribution of each character to the story.

**Fig 5:**
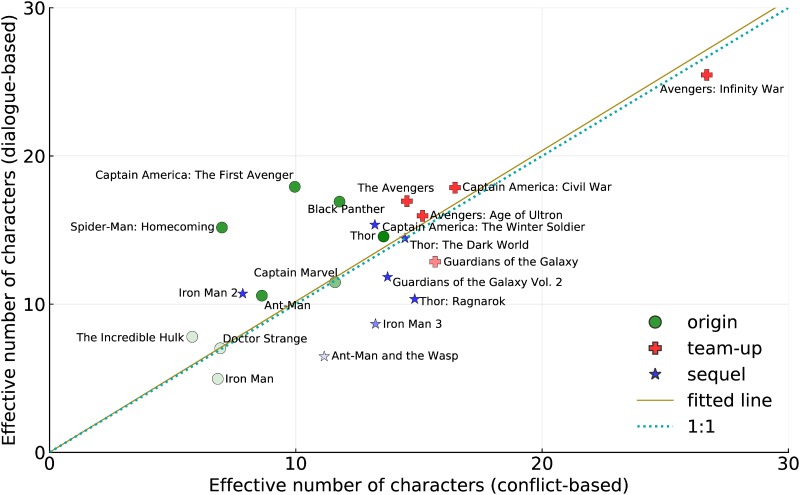
Conflict and dialogue metrics of cast size. Darker shading indicates complete (dialogue) datasets; paler shading indicates incompleteness.

Fig 5 shows a scatter plot of the two alternative metrics. The plot also shows two reference lines: the first a fit (through zero) to the data, and the second a 1:1 line. The former shows that overall the dialogue measures of cast size report very slightly larger numbers than the conflict based metric. They are, on average, remarkably close given the fact the two use completely different views of the movie.

However, there are additional patterns of note. We can understand that movies that sit above the reference line contain more dialogue-based participation, and less conflict, and in turn, those below the line entail more conflict. Extreme examples are *Spider-Man* and *Captain America: The First Avenger* (which are dialogue heavy) and *Thor: Ragnarok* (which is action dominated).

Origin movies usually lie above the line (or close to it). Origin movies often use extensive dialogue to show character development from “zero to hero.”

Sequels seem, on the other hand, to lie close to or below the line, thus involving more action than dialogue. Team-ups often lie quite close to the line: the formation of a team often requires the members to meet and talk, but these movies use action to display the abilities of the entire team.

The location of a movie in this plot represents subtle changes in style in screenplay and direction. It is interesting that a level of detail that one might expect only to be exposed by detailed semantic analysis of content can be seen in a single pair of metrics.

## Movie comparison results

An additional task for which cast metrics are useful is comparison of movies. That is, we would like to have a metric for how similar or dissimilar two movies are. For the same reasons that we consider above, it would be useful to have a metric that is measured in units that correspond to effective cast size, *i.e*., it would be appealing to say that the difference between two movies is *X* effective cast members. Thus we should have that two disjoint movies *A* and *B* would have an distance that is directly related to $\hat{N}\left(A\right)+\hat{N}\left(B\right)$, and that two movies with identical character contributions would have a distance of 0.

There are many formal distance metrics that can be applied to probability distributions (in this section we use the conflict-based distributions). A survey of metrics and their properties can be found in [49]. Typical distance metrics between probability distributions either

1. assume the same support, *i.e*., that the two have positive support on an identical set of elements (*e.g*., Kullback-Leibler or *χ*^2^ divergences), or
2. they ignore the measures on the elements and simply use counts of the size or relative size of overlaps of support (*e.g*., Jaccard [50] or Sørensen–Dice [51, 52] coefficients).

Neither of these two conditions is applicable in our domain. We need a metric that takes into account the probabilities, but also allows the supports to vary because many movies have different casts.

There are two entropy-based approaches we can adopt: one formal, and the other more intuitive. The formal approach is to adapt the Jensen–Shannon divergence (JSD) [53, 54] (also called the *total divergence to the average* or the *increment of the Shannon entropy*). This is a symmetrised, smoothed version of the Kullback–Leibler divergence (KLD). It can be defined in terms of the KLD

$$\begin{array}{c} D_{KL}\left(P\|Q\right)=-\sum_{x\in X}P\left(x\right)\;\text{log}\;\frac{Q(x)}{P(x)}, \end{array}$$

as

$$\begin{array}{c} D_{JS}\left(P\|Q\right)=\frac{1}{2}[D_{KL}\left(P\|M\right)+D_{KL}\left(Q\|M\right)], \end{array}$$

where *M* = (*P* + *Q*)/2 is short-hand for the distribution formed from the averages of the probabilities of the two distributions or equivalently in terms of entropy

$$\begin{array}{c} D_{JS}\left(P\|Q\right)=H(M)-\frac{1}{2}[H(P)+H(Q)]. \end{array}$$

It has the advantage of being theoretically understood [53, 54], *e.g*., we know that 0 ≤ *D*_*JS*_(*P*‖*Q*) ≤ 1. In linguistics [53] this has been used by taking an exponent, the natural such for us being to take a power of 2, *i.e*.,

$$\begin{array}{c} {\bar{D}}_{JS}=2^{D_{JS}\left(P\|Q\right)}-1. \end{array}$$

We subtract 1 because we should like our measure of dissimilarity to be 0 when comparing a movie to itself. We can form a measure of similarity then by noting that the maximum value of *D*_*JS*_ is 1, and taking

$$\begin{array}{c} {\bar{S}}_{JS}=1-{\bar{D}}_{JS}. \end{array}$$

The approach above is theoretically appealing, consistent with our use of entropic measures, and useful, but it lacks an interpretation in terms of effective cast size. That is, we can only interpret ${\bar{S}}_{JS}$ as describing the *proportion* of cast members in common.

An alternative measure is to proceed with the calculation we might pursue in terms of measuring the effective increase in cast size of the combination of two movies over the average size of the casts by taking

$$\begin{array}{ccc} D_{\text{intuitive}}\left(P\|Q\right) & = & \hat{N}\left(M\right)-\text{average}[\hat{N}\left(P\right)+\hat{N}\left(Q\right)]\\ & ≃ & 2^{H(M)}-2^{(H(P)+H(Q))/2}. \end{array}$$

This expression looks very similar to that for ${\bar{D}}_{JS}$ but it has units of characters.

The two appear similar, and in fact are very closely related if we normalise *D*_intuitive_, by taking

$$\begin{array}{c} {\bar{D}}_{\text{intuitive}}\left(P\|Q\right)=\frac{D_{\text{intuitive}}\left(P\|Q\right)}{(\hat{N}\left(P\right)+\hat{N}\left(Q\right))/2} \end{array}$$

and as before we form a similarity measure ${\bar{S}}_{\text{intuitive}}=1-{\bar{D}}_{\text{intuitive}}$.

We will contrast these with several other standard measures used in ecology to measure the difference between populations in different regions:

- The Jaccard index [50] (Jaccard called his index a *coefficient of community*) and it is defined by
  $$\begin{array}{c} S_{J}\left(P,Q\right)=\frac{\left|\text{supp}(P)\cap \text{supp}(Q)\right|}{\left|\text{supp}(P)\cup \text{supp}(Q)\right|}, \end{array}$$
  where supp(·) denotes the support of the distribution, *i.e*., the set of characters that appear in the movie, The corresponding distance is *D*_J_(*P*, *Q*) = 1 − *S*_J_(*P*, *Q*).
- The Sørensen–Dice coefficient [51, 52] defined by
  $$\begin{array}{c} S_{\text{SD}}\left(P,Q\right)=\frac{2|\text{supp}(P)\cap \text{supp}(Q)|}{\left|\text{supp}(P)|+|\text{supp}(Q)\right|}, \end{array}$$
  whose corresponding dissimilarity *D*_SD_(*P*, *Q*) = 1 − *S*_SD_(*P*, *Q*) is often called the Bray-Curtis dissimilarity [55].
- Euclidean distance
  $$\begin{array}{c} D_{\text{SD}}\left(P,Q\right)=\sqrt{\sum_{i}{\left(p_{i}-q_{i}\right)}^{2}}. \end{array}$$

There are many other such distances, but these suffice to show the various types of approaches, showing both the set-based and distance based approaches. Others often correspond to transformations or re-weightings of these approaches.

We test the different metrics quantitatively on their clustering performance. A good metric should allow us to cluster the movies naturally. We break the movies into 11 clusters according to the major strands of the franchise as shown in Table 7.

**Table 7:** Clusters used in the quantitative assessment of the dissimilarity metrics.

Title	Group label
Ant-Man	1
Ant-Man And The Wasp	1
Avengers: Age Of Ultron	2
Avengers: Infinity War	2
The Avengers	2
Black Panther	3
Captain America: Civil War	4
Captain America: The First Avenger	4
Captain America: The Winter Soldier	4
Captain Marvel	5
Doctor Strange	6
Guardians Of The Galaxy	7
Guardians Of The Galaxy Vol. 2	7
Iron Man	8
Iron Man 2	8
Iron Man 3	8
Spider-Man: Homecoming	9
Thor	10
Thor: Ragnarok	10
Thor: The Dark World	10
The Incredible Hulk	11

We compare the different clustering approaches through standard measures of their accuracy, in particular the precision, recall, Rand metric and F1 score. The results are shown in Table 8. All confirm that the clustering performance of the intuitive metric is superior to the alternatives, with the Jensen-Shannon metric performing second best by most measures.

**Table 8:** Clustering performance for the different dissimilarity metrics.

Dissimilarity	Precision	Recall	Rand	F1 score
Intuitive	0.824	1.0	0.986	0.903
Jensen-Shannon	0.609	1.0	0.957	0.757
Jaccard	0.625	0.714	0.952	0.667
Bray-Curtis	0.625	0.714	0.952	0.667
Euclidean	0.382	0.929	0.895	0.542

A natural question is why the performance of the intuitive approach is superior. With respect to Jaccard and Bray-Curtis this is because the intuitive metric uses more information, namely the proportionate participation. The Euclidean method performs badly because it does not appreciate that a difference between two important characters, and two unimportant characters should not have the same weight. It is possible to re-weight the Euclidean metric, however, the better weightings tend to be those that are in actuality approximations to entropy-related measures.

**Fig 6:**
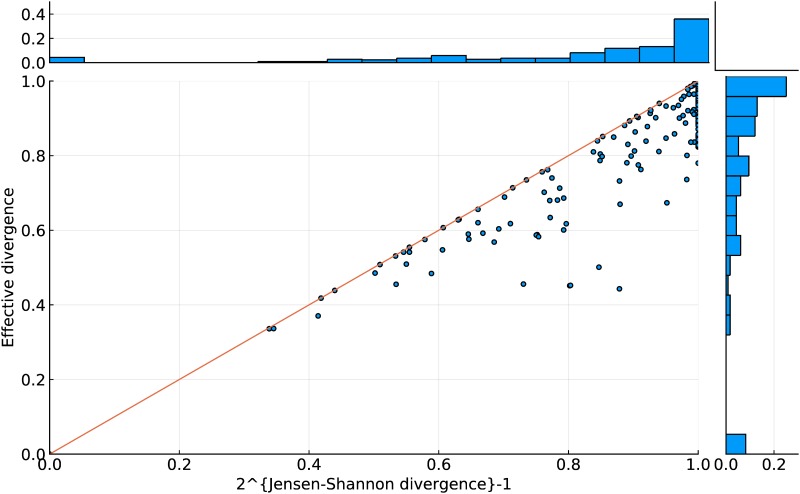
A comparison of the two entropy-based distance metrics showing that the intuitive divergence ${\bar{D}}_{\text{intuitive}}$ lies below the Jensen-Shannon measure ${\bar{D}}_{JS}$ (each point corresponds to one pair of movies).

These types of problems with simplistic metrics are not unknown in the ecological setting. The result that is perhaps surprising however is that the novel intuitive measure performs better than the Jensen-Shannon metric. One might expect their performance to be very similar as they are based on the same underlying concept. Fig 6 shows a comparison between the two. The intuitive measure is highly correlated with the Jensen-Shannon measure, but has the advantage that the large mass of cases with ${\bar{D}}_{JS}=1$ are spread out over a range of values of ${\bar{D}}_{\text{intuitive}}$ apparently providing a little more information of use in clustering.

It would be very interesting to repeat this analyses on other movie franchises, but there are very few that have the scale and complexity of the MCU. The Bond series, for instance, has a very small set of characters that are repeated in almost every movie.

**Fig 7:**
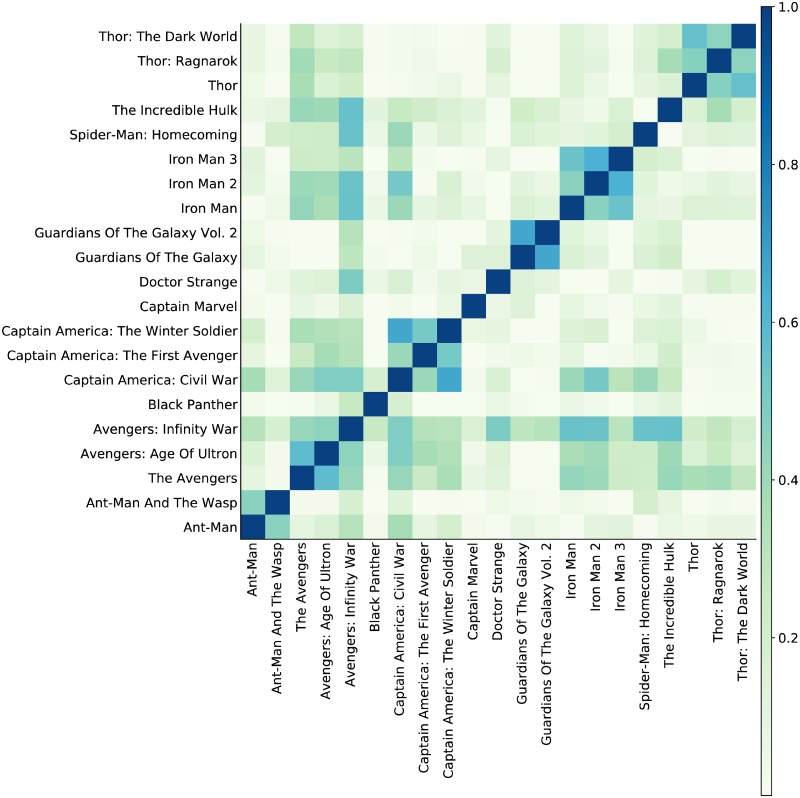
Heat map of normalised similarities ${\bar{S}}_{\text{intuitive}}$ between pairs of movies. Evident are blocks of movies corresponding to the major sub-sequences, *e.g*., the Thor movies or the Iron Man movies. Also noticeable is the sharing of cast between the Avengers (team-up) movies and many of the others.

**Fig 8:**
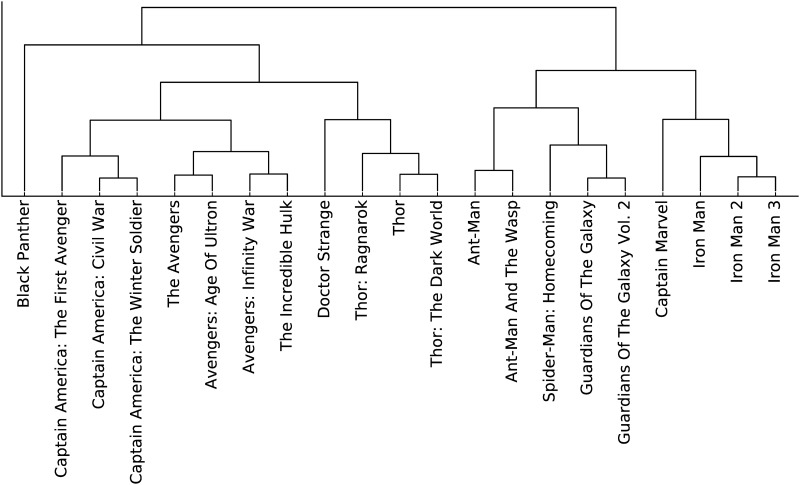
Dendrogram derived from hierarchical clustering of the movies based on the dis-similarities *D*_*A*,*B*_.

Fig 7 shows a heat-map of the similarities ${\bar{S}}_{\text{intuitive}}$ to further highlight the blocks of clustered movies, and we can draw out these clusters, for instance, using the dendrogram presented in Fig 8.

**Fig 9:**
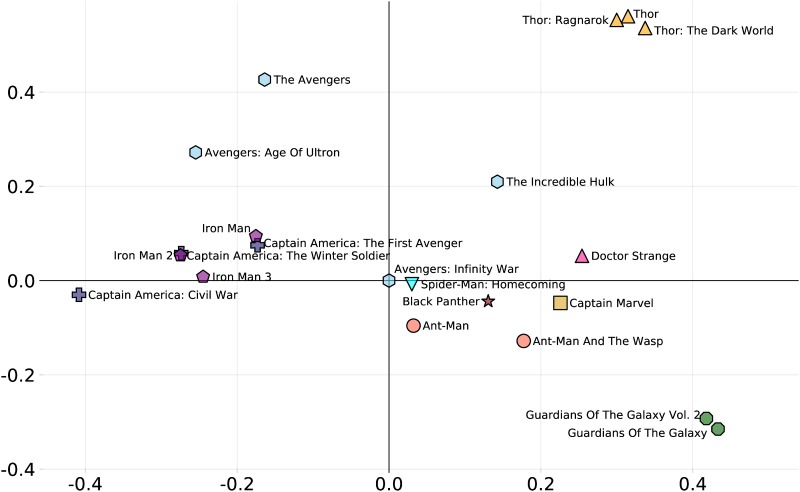
MDS projection into a 2D space based on the cast dissimilarities. Note that a small translation has been applied to place *Avengers: Infinity War* at the origin.

We further illustrate the intuitive dissimilarity by performing (classical) MDS on the intuitive dissimilarity matrix *D* to obtain a projection of the movies into local coordinates in a 2D Euclidean space. The coordinates are shown in Fig 9, which also shows the clustering results. The original metric space being embedded is highly non-Euclidean (many of the distances are near 1), and so the 2D embedding is a poor approximation in some ways, but it does allow visualisation of the clusters.

Of note:

- the upper-right quadrant is the Thor quadrant (the *Incredible Hulk* falls into this region because the Hulk plays a major role in *Thor: Ragnarok*.
- the upper-left quadrant is an Avengers/Iron Man/Captain America cluster. These movies are tightly wound together so it is no surprise that they appear together in this quadrant.
- The lower half holds a more miscellaneous collection of movies, though the two Guardians movies cluster tightly.

Note that although the Captain America movies are in a separate cluster with the Iron Man/Avengers cluster, they appear to overlap in the plot. This illustrates the limitations of projecting into a 2D space, the 3D projection more clearly separates these clusters, but is hard to illustrate here.

Informally, we can see in the plot a left-to-right division between the more technology-derived superheros (Iron Man and Captain America) vs those that come from a magical or alien background (Doctor Strange, Thor and the Guardians). The placement of the Hulk and Ant-Man towards the right is somewhat of a surprise from this point of view, but perhaps reflects that sometimes superpowers are really “magic” explained as technology. Alternatively we might see the left-right axis as separating the more rationalist heroes from the more emotional heroes. In terms of personality classification (*e.g*., Myers-Briggs) we might see the left-hand side of the chart as relating to *thinking* and the right to *feeling*. We can see characters such as Iron Man/Tony Stark and Captain America/Steve Rogers as both making reasonable (if different) decisions based on tangible evidence and codified, consistent sets of rules. On the other hand, characters such as Star-Lord/Peter Quill from the Guardians and Thor often act intuitively or emotionally (sometimes illogically from the perspective of other characters).

## Conclusion and future work

Measuring the number of characters in the a movie or franchise such as the Marvel Cinematic Universe is an interesting and novel challenge. While naively seen as easy, there is a need for a robust metric of character count that incorporates character participation. Such a metric is less sensitive to noise in the input data, and harder for a commercial entity to “game.”

The use of Shannon-entropy based measures built from frequency distributions of participation provides a metric based on ecological diversity, which is intuitive, robust and useful. The robustness is shown through the comparisons of the metric under constructions from dialogue and visual conflicts separately, and alternative transcriptions of the same films. The usefulness of this metric is explored with respect to the success of the movies measured via their profitability and shown to have a strong correlation. A generalisation of this metric using Jenson-Shannon divergence allows a measure of similarity between movies that gives rise to a clean clustering of movies, supporting the notion of usefulness of this metric as a tool for the taxonomy of movies, at least in the MCU.

The usefulness of this metric does not end in the Marvel Universe and possible extensions of this work are widespread. The presented metric and its generalisation could be explored in relation to other cinematic universes (*e.g*., DC vs Marvel), or movie genres, TV shows or other forms of media entirely, *e.g*., comics. Further, the metric has been applied to the Shannon-entropy of frequency distributions, however, this metric could be applied to a variety of other sources with relevance to character sentiments, transitions between evil and good characters, or the entropy of the speaker sequence as a Markovian process. It could even be applied to locations or objects appearing in the narrative, as such can be quite important in classifying, for instance, folk stories.

We speculate that the uses might extend even beyond fiction. For instance, increases in the number of authors on scientific papers over time have sometimes been observed [56]. It is becoming more common for authors to list in some way their contribution to the paper, and so a metric such as that proposed here this could provide effective measures of the numbers of authors on a paper that take into account that all do not contribute equally. We might thereby understand if these inflated author counts are real, or an artefact of changes in scientific publication standards.

## Supporting information

S1 Fig The numbers of credited characters in the MCU movies. (EPS)

S2 Fig The numbers of named characters in the MCU movies. (EPS)

S3 Fig Jenson-Shannon similarities (compare to Fig 7). (EPS)
